# Structural MRI Biomarkers of Intracranial Pressure in IIH: Linking Optic Nerve Sheath, Pituitary Morphology, and Hormonal Changes

**DOI:** 10.1007/s00062-026-01618-8

**Published:** 2026-01-27

**Authors:** Zeynep Bendella, Barbara Daria Wichtmann, Ralf Clauberg, Wiebke Fenske, Charlotte Fries, Monika Jeub, Martina Minnerop, Arndt-Hendrik Schievelkamp, Franziskus M. Schützeichel, Bettina Wabbels, Christina Schaub, Max Witry, Berkan Koyak, Alexander Radbruch, Jennifer Linder, Ullrich Wüllner, Christine Kindler

**Affiliations:** 1 Departement of Neuroradiology, University of Bonn, Bonn, Germany; 2 Clinical Neuroimaging Group, German Center for Neurodegenerative Diseases, Bonn, Germany; 3 Department of Medicine, Endocrinology and Diabetes, BG University Hospital Bergmannsheil Bochum, Bochum, Germany; 4 Division of Endocrinology, Diabetes and Metabolism, Department I for Internal Medicine, University of Bonn, Bonn, Germany; 5 Department of Neurology, University of Bonn, Bonn, Germany; 6 Institute of Neuroscience and Medicine (INM-1), Research Center Juelich, Juelich, Germany; 7 Institute of Clinical Neuroscience and Medical Psychology, Heinrich Heine University Düsseldorf, Düsseldorf, Germany; 8 Department of Nuclear Medicine, University Hospital Cologne, Cologne, Germany; 9 Department of Ophthalmology, University of Bonn, Bonn, Germany; 10 Department of Neurology, University Hospital Knappschaftskrankenhaus Bochum, Bochum, Germany; 11 Department of Neurooncology, Center of Neurology, University of Bonn, Bonn, Germany; 12 German Society of Intracranial Hypertension, Bad Honnef, Germany; 13 Department of Parkinson, Sleep and Movement disorders, University of Bonn, Bonn, Germany; 14 Department of Vascular Neurology, University of Bonn, Bonn, Germany; 15 Department of Neurology, Center for Movement Disorders and Neuromodulation, Heinrich-Heine-University Düsseldorf, Düsseldorf, Germany

**Keywords:** Idiopathic intracranial hypertension, Optic nerve sheath, Hormone axes, Growth hormone, Thyroid stimulating hormone, Intracranial pressure

## Abstract

**Purpose:**

To investigate the relationship between intracranial pressure (ICP), anterior pituitary hormones, and structural brain changes in women with idiopathic intracranial hypertension (IIH).

**Methods:**

Eighteen women with therapy-refractory IIH underwent lumbar puncture, endocrine assessment, and high-resolution brain MRI. Serum levels of pituitary hormones were correlated with ICP and radiological parameters including pituitary volume, flattening, and optic nerve (ON) and optic nerve sheath (ONS) volume. Group comparisons and partial correlations were used to evaluate associations.

**Results:**

ICP showed a significant positive association with thyroid-stimulating hormone (TSH) levels (r = 0.628, *p* = 0.016), and a significant negative association with growth hormone (GH) (r = −0.602, *p* = 0.023). Regarding structural parameters, only the volume of the right ON showed a strong positive association within the subgroup with elevated ICP (r = 0.90, *p* = 0.005). Correlations between ONS volumes and ICP in the normal pressure subgroup narrowly missed statistical significance. TSH was the only hormone showing a significant association, with higher TSH levels relating to larger pituitary volume in the normal ICP subgroup (r = 0.88, *p* = 0.020), but not in the elevated ICP subgroup.

**Conclusion:**

Our exploratory findings suggest potential interactions between ICP, endocrine markers, and structural MRI measures. However, due to the limited sample size and variability in endocrine parameters, the results should be interpreted cautiously and considered hypothesis generating rather than clinically directive. Larger studies are needed to determine whether endocrine MRI associations hold true and whether they have diagnostic or clinical relevance.

## Introduction

Idiopathic intracranial hypertension (IIH) is characterized by elevated intracranial pressure (ICP) without a detectable structural lesion or hydrocephalus. IIH primarily affects obese women of childbearing age and carries a risk for severe symptoms, including vision loss [1, 2]. The underlying mechanisms of IIH remain incompletely understood, although multifactorial contributions have been proposed, including alterations in cerebrospinal fluid (CSF) dynamics, venous outflow obstruction, and hormonal dysregulation [3, 4].

While the association between IIH and obesity is well-established, accumulating evidence suggests that endocrine abnormalities may also contribute to its pathophysiology.

Several case reports and small cohort studies [5–8], as well as more recent observational work, have described associations between IIH and hormonal imbalances involving the thyroid, adrenal, gonadal, and somatotropic axes [3, 9–12]. In particular, thyroid dysfunction has been disproportionately reported in IIH, and recent evidence suggests normalization of thyroid-stimulating hormone (TSH) levels following ICP reduction [12]. Nevertheless, systematic endocrine investigations in relation to ICP remain scarce, particularly in adults.

In parallel, structural MRI findings such as an empty sella, pituitary flattening, and optic nerve sheath (ONS) distension are frequently observed in IIH and are commonly attributed to mechanical effects of elevated ICP [13, 14]. However, it remains unclear whether these changes reflect true endocrine dysfunction or merely passive compression. Prior studies have reported hypopituitarism in patients with empty sella in up to 52% of cases, with growth hormone (GH) deficiency particularly prevalent [15].

Given the bidirectional interactions between hormonal function and CSF physiology-including evidence for the expression of hormone receptors and transporters in the choroid plexus and arachnoid granulations [16–18], an integrated analysis of structural and endocrine markers may offer new insights into IIH.

In this study, we investigated the relationship between anterior pituitary hormone levels and quantitative MRI measures in women with IIH. We explored whether elevated ICP is associated with alterations in the thyroid and somatotropic axes and that these endocrine changes may be accompanied by structural brain alterations detectable on MRI. By integrating hormonal data with volumetric imaging markers such as pituitary gland volume, pituitary flattening, ON and ONS volume, we aim to refine the understanding of neuroendocrine regulation in IIH and to evaluate potential non-invasive markers of disease burden.

To test these hypotheses, we conducted a cross-sectional study in a well-defined cohort of women with therapy resistant IIH. We systematically assessed anterior pituitary hormone levels and performed high-resolution brain MRI to quantify structural changes. We statistically examined associations between hormone levels, imaging features, and ICP measured via lumbar puncture.

## Materials and Methods

### Study Design and Participants

In this cross-sectional study, we consecutively enrolled 18 female patients with therapy refractory IIH who were hospitalized due to recurrent symptoms requiring lumbar puncture between May 2018 and October 2019. The diagnosis of IIH was confirmed according to the modified Dandy criteria [19] by reviewing external medical reports for individual diagnostic components. Exclusion criteria included age under 18 years, neurological disorders other than IIH, contraindications to MRI (e.g., history of seizures, severe psychiatric or systemic illness, metallic implants, claustrophobia, pregnancy), and substance abuse. Sample size was based on data availability; no power calculation was performed. All subjects provided written informed consent, and the study was approved by the local ethics committee (No. 383/17), conducted in accordance with the Declaration of Helsinki.

Demographic and clinical variables included age, body mass index (BMI), waist-to-hip ratio, medication use, and detailed neurological, gynecological, and ophthalmological history. Polycystic ovary syndrome (PCOS) was diagnosed using the Rotterdam criteria [20], requiring at least two of the following: oligo- or amenorrhea, clinical or biochemical hyperandrogenism, or polycystic ovaries on ultrasound.

### Endocrinological Assessment

Venous blood samples were collected from all subjects after overnight fasting at 7:00 am. Each blood sample was processed according to the standard protocols of our clinical laboratory.

Post hoc serum concentrations of thyroid hormones, including TSH, free thyroxine (fT4), and triiodothyronine (fT3), gonadotropins, including follicle stimulating hormone (FSH) and luteinising hormone (LH), as well as reproductive hormones, such as estradiol, testosterone, androstenedione, and dehydroepiandrosterone sulphate (DHEAS), were analysed. Glucocorticoids adrenocorticotropic hormone (ACTH) and cortisol, as well as GH, were measured using an electrochemiluminescence immunoassay (ECLIA) (Roche Diagnostics). Serum concentrations of insulin-like growth factor 1 (IGF-1) were determined using an Immunodiagnostic Systems (IDS®) immunoassay. Levothyroxine intake was considered indicative of hypothalamic-pituitary-thyroid-axis dysregulation regardless of TSH levels. Values below detection thresholds were set to half the detection limit. Prolactin was measured but excluded from inferential analyses because its secretion shows rapid stress-related fluctuations, including venipuncture-induced hyperprolactinaemia, and lacks a stable anatomical relation to pituitary size [21–23].

### Lumbar Puncture

Lumbar puncture was performed in lateral decubitus position on the same day as the endocrine evaluation. ICP was measured in cmH2O. If ICP was ≥ 25 cmH2O, 20–30 mL of CSF were drained for therapeutic relief. Based on ICP measurements, patients were categorized into a normal (< 25 cmH2O) and elevated (≥ 25 cmH2O) group. Given the small subgroup sizes, this dichotomization was used exclusively for descriptive and exploratory comparisons.

### Ophthalmological Examination

Ophthalmological examination included assessments of the optic disc, visual field, visual acuity, and bulbar motility. Papilledema was assessed using fundus examination and the Frisén scale [24], graded from 0 to 5. Visual acuity was measured with the revised Snellen chart, with reduction defined as < 20/25. Visual fields were assessed using the OCULUS Twinfield perimeter with automated kinetic (stimulus III/4) and static perimetry (program 30‑2, stimulus size III, fast threshold strategy). The mean deviation (MD), indicating total field loss, was automatically generated. Field restriction was defined as < 40° nasal or < 75° temporal. Enlarged blind spots were also assessed. Visual fields with unacceptable reliability indices (loss of fixation > 30%, false-positive errors > 15%) were excluded. Optic disc volume was measured using optical coherence tomography (OCT) with Spectralis® (Heidelberg Engineering, Heidelberg, Germany).

### Imaging Protocol and Morphometric Analysis

High-resolution structural brain MRI was performed before lumbar puncture to exclude secondary causes of raised ICP. For morphometric analysis, volumetric T1-weighted images were used to segment and quantify pituitary gland volume, sella turcica volume, pituitary gland flattening (defined as percentage of sella filled), left and right ONS and optic nerve (ON) volumes each.

**Fig. 1:**
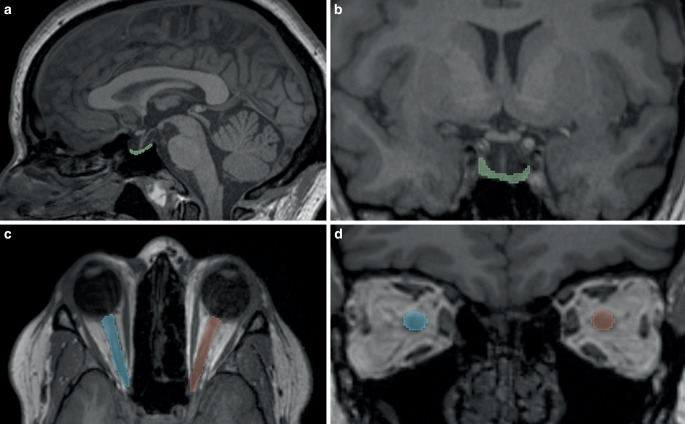
Voxel-based segmentation of the pituitary gland and optic nerve sheath (ONS) in a representative patient with idiopathic intracranial hypertension (IIH). **a** Midsagittal T1-weighted MRI scan showing the segmented pituitary gland (green). **b** Coronal T1-weighted scan illustrating pituitary segmentation (green) in the frontal plane. **c** Axial T1-weighted scan with bilateral segmentation of the ONS (blue: left; red: right). **d** Coronal T1-weighted scan showing ONS segmentation (blue and red) in the transverse plane. These images illustrate typical morphological changes in IIH and the applied voxel-based approach to structural quantification

Segmentations were performed manually using 3D Slicer (version 5.8.0) by an experienced neuroradiologist blinded to ICP group. Incomplete empty sella was defined as 0–50% CSF-filling, and complete empty sella as > 50%. Empty sella classification was recorded descriptively and not used to infer pituitary function. Intra-rater reliability was confirmed by duplicate measurement across all participants. Representative segmentations are shown in Fig. 1.

### Statistical Analyses

Statistical analyses were performed in Python (v3.11.8) using NumPy (v1.26) and pandas (v2.1) for data handling, SciPy.stats (v1.11) for distribution testing and group comparisons, and pingouin (v0.5) for partial correlation analyses. Figures were generated in Python using matplotlib (v3.8) for plotting and seaborn (v0.13) for regression lines and confidence intervals.

Descriptive statistics were reported as means with standard deviations for normally distributed variables and as medians with interquartile ranges for non-normally distributed variables. Normality was assessed using the Shapiro Wilk test. Baseline group differences in demographic, clinical, and imaging characteristics were assessed using Student’s t‑test or Mann-Whitney U‑test for continuous variables and chi-squared or Fisher’s exact tests for categorical variables. Effect sizes (Cohen’s d, rank biserial correlation, Cramer’s V, or Phi coefficient) were reported for all group comparisons. Missing values were excluded pairwise, and the resulting sample sizes are noted in the tables.

Subsequently, we conducted partial correlation analyses to examine (i) associations between ICP and structural imaging measures (pituitary volume, pituitary flattening, sella volume, and bilateral ONS and ON volumes), (ii) associations between pituitary hormone levels (TSH, LH, FSH, ACTH, GH) and pituitary structure, and (iii) associations between ICP and the full endocrine panel comprising pituitary hormones (TSH, LH, FSH, ACTH, GH) and peripheral hormones (fT3, fT4, DHEAS, testosterone, estradiol, androstenedione, cortisol).

All subsequent analyses were performed in the full cohort and repeated within ICP defined subgroups. Partial correlations were selected to quantify bivariate associations while controlling for predefined covariates. Pearson partial correlations were applied when residuals approximated a normal distribution. Spearman partial correlations were used otherwise. Age and BMI were included as covariates in all correlation models based on their known influence on ICP regulation, body composition, and endocrine physiology [25–27]. These variables were chosen a priori and were included to reduce physiological variance rather than to imply causality.

For analyses assessing the relationship between endocrine parameters and ICP, pituitary gland volume was included as an additional covariate to account for interindividual anatomical variation that may influence circulating hormone concentrations. The adjustment follows prior work reporting associations between pituitary size and gonadotropin levels, although published findings remain inconsistent [28–30]. Given the small sample size, the analytic strategy was restricted to partial correlations and simple regression to minimize the risk of model overfitting. For visualization, covariate-adjusted scatterplots were generated using residuals from the corresponding regression models; these residuals do not represent absolute hormone concentrations but depict covariate-adjusted relationships for illustrative purposes.

A sensitivity analysis performed in G*Power 3.1 showed that with *n* = 18, α = 0.05, and power = 0.80, only large correlations (|r| ≈ 0.47 or greater) could be detected inconsistent [31, 32]. Correlation results were therefore interpreted as exploratory and hypothesis-generating. All statistical tests were two-sided with a significance threshold of *p* < 0.05.

## Results

### Demographic and Clinical Profile

Eighteen women with therapy-refractory IIH were enrolled between May 2018 and October 2019 (Table 1). Ten patients showed elevated ICP (≥ 25 cmH_2_O; median 26.5 cmH_2_O, IQR 25.3–31.0), while eight had normal pressures (mean 19.0 ± 2.4 cmH_2_O). Patients with elevated ICP were slightly older and had higher BMI values compared with those with normal ICP, although these differences were not statistically significant (Table 1). Waist to hip ratios were comparable across groups. Extended baseline clinical, imaging, ophthalmologic, and endocrine characteristics including PCOS prevalence, neurological comorbidities, medication use, and OCT derived optic disc metrics showed no relevant group differences and are summarized in Supplementary Table S1–S3.

**Table 1:** Demographic and clinical group characteristics for the total group and for normal and elevated ICP. Independent variable: ICP group (normal < 25 cmH_2_O vs. elevated ≥ 25 cmH_2_O)

	Whole Group	ICP < 25 cmH_2_O	ICP ≥ 25 cmH_2_O	*P* Value	Test Statistics	Cohen’s d/Rank-Biserial/Phi coefficient (φ)/Cramer’s V
Age [years]^a^	35.0 ± 8.2 (*n* = 18)	31.9 ± 7.2 (*n* = 8)	37.5 ± 8.4 (*n* = 10)	0.152	t(16) = −1.505	−0.714
BMI [kg/m2]^a^	35.3 ± 6 (*n* = 18)	33.1 ± 5.3 (*n* = 8)	37.0 ± 6.2 (*n* = 10)	0.180	t(16) = −1.401	−0.665
Waist to Hip ratio^a^	0.8 ± 0.1 (*n* = 18)	0.8 ± 0.1 (*n* = 8)	0.8 ± 0.1 (*n* = 10)	0.549	t(16) = 0.612	0.626
ICP [cmH_2_O]^b^	24.3 ± 6.0 (*n* = 18)	19.0 ± 2.4 (*n* = 8)	26.5 [25.3, 31.0] (*n* = 10)	*p* *<* 0.001*	U = 0.000	1

Group characteristics are presented as mean ± standard deviation (SD) for normally distributed variables, as median (interquartile range (IQR)) for non-normally distributed variables. Depending on distribution, either the independent Student’s t‑test (a), the Mann-Whitney U test (b) was applied. Corresponding effect sizes (Cohen’s d or Rank Biserial correlation) are reported for each comparison. Statistical significance was set at *p* < 0.05 two-sided. BMI = Body Mass Index; cmH_2_O = centimeter water column; ICP = intracranial pressure.

### Structural MRI Findings and ICP

Exploratory analysis indicated that a significant association between ICP and the volume of the right ON was present in the subgroup with elevated ICP (r = 0.90, *p* = 0.005). No corresponding relationship was observed in the group with normal ICP (r = 0.12, *p* = 0.82). None of the remaining structural measures, including pituitary volume, optic nerve volume, or optic nerve sheath metrics, showed significant associations with ICP in the full cohort or within either subgroup (Supplementary Table S4).

For ONS volumes, correlation coefficients in the normal ICP subgroup were relatively high and *p*-values narrowly missed significance. These near threshold trends may reflect variation within the lower ICP range, but the data do not provide evidence for a robust association.

### Endocrine Correlates of Pituitary Morphology

**Fig. 2:**
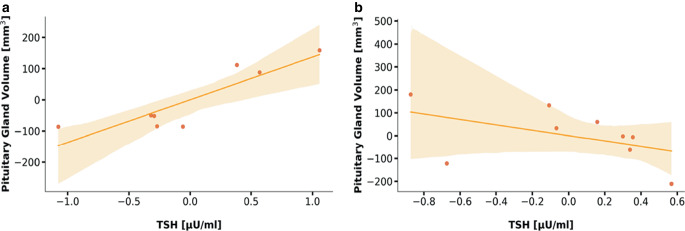
Scatterplots with regression lines illustrating the association between serum TSH levels (μU/mL) and pituitary gland volume (mm^3^), shown separately for **a** patients with normal ICP (< 25 cmH_2_O) and **b** patients with elevated ICP (≥ 25 cmH_2_O). Hormone values are adjusted for age and BMI, the plotted values represent model residuals rather than absolute physiological concentrations, which can result in negative values as a statistical artefact. Shaded areas indicate 95% confidence intervals around the regression line. Abbreviations: cmH_2_O = centimeter water column; *ICP* intracranial pressure; *mm*^*3*^ cubic millimeter; *TSH* thyroid-stimulating hormone

A significant positive association between TSH and pituitary volume was observed only in the normal ICP group (r = 0.88, *p* = 0.020; Fig. 2a). No corresponding relationship was present in the elevated ICP group (Fig. 2b). No significant associations for LH, FSH, ACTH, or GH were detected in either subgroup (Supplementary Table S5).

### Endocrine Associations with ICP

**Fig. 3:**
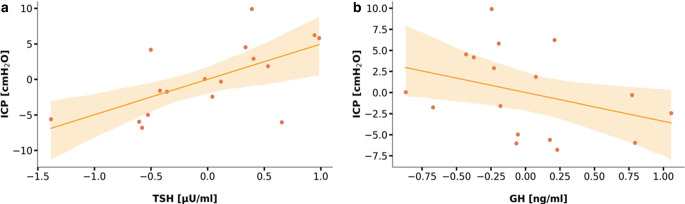
Scatterplots with regression lines illustrating the relationship between ICP (cmH_2_O) and serum hormone levels in IIH for **a** TSH and **b** GH (ng/mL), adjusted for age, BMI, and pituitary gland volume. Adjusted values represent model residuals rather than absolute physiological concentrations and may therefore take negative values. Shaded areas indicate 95% confidence intervals around the regression lines. *cmH*_*2*_*O* centimeter water column; *GH* growth hormone; *ICP* intracranial pressure; *TSH* thyroid-stimulating hormone

ICP was positively associated with TSH (r = 0.628, *p* = 0.016) and negatively associated with GH (r = −0.602, *p* = 0.023; Supplementary Table S6; Fig. 3). No other endocrine parameter showed a significant association with ICP.

## Discussion

This study provides preliminary observations on associations between elevated ICP, anterior pituitary hormone alterations, and structural brain changes in women with IIH. Through the integration of endocrine profiling and quantitative MRI, we observed coherent patterns suggesting that sustained elevations in ICP may influence both thyroid and somatotropic regulation while also affecting optic nerve morphology. Although these findings require cautious interpretation, they provide preliminary insight into the potential neuroendocrine involvement in IIH.

Our findings highlight the thyroid axis as one hormonal system that may be particularly sensitive to elevated ICP. TSH levels showed a significant positive correlation with ICP (r = 0.628, *p* = 0.016), and subgroup analyses provided additional insight. In individuals with normal ICP, TSH demonstrated a strong positive association with pituitary volume (r = 0.882, *p* = 0.020), whereas no comparable relationship was present in the elevated ICP group (r = −0.478, *p* = 0.278). This divergence may reflect preserved glandular responsiveness under normal pressure conditions, with reduced volume hormone coupling in the context of sustained ICP elevation. The effects detected in our cohort emerged despite the limited sample and a sensitivity analysis indicating that only relatively large correlations (approximately |r| ≥  0.47) could be reliably observed, which implies that smaller endocrine effects may not have been detected.

Prior cohort studies have reported inconsistent findings regarding thyroid involvement in IIH. While Giuseffi et al. [2] described an identical prevalence of thyroid disease in IIH patients and controls (2%), KesKin et al. [33] reported a substantially higher combined rate of diabetes and thyroid disease (19%) in IIH. More recently, Prabhat et al. [12] observed normalization of elevated TSH following ICP reduction, further supporting a potential bidirectional interaction between ICP and thyroid regulation. In our cohort, the prevalence of thyroxine supplementation was higher in patients with elevated ICP (30% vs. 13%), exceeding population estimates of thyroid hormone use (5–12%) [34–36]. Although prior reports have suggested increased thyroid vulnerability in selected IIH populations [5–8, 37–39], this difference may also reflect demographic characteristics such as female predominance and elevated BMI [40, 41]. Biological plausibility is supported by experimental work demonstrating expression of TSH receptors and thyroid hormone transporters in the choroid plexus, indicating that ICP-dependent influences on thyroid regulatory pathways are biologically conceivable [16, 18, 42, 43]. Taken together, the available evidence suggests a potential interaction between ICP and the thyroid axis, although neither causality nor clinical implications can be inferred from our dataset.

The somatotropic axis showed an inverse pattern, with serum GH levels demonstrating a negative correlation with ICP (r = −0.602, *p* = 0.023). Previous reports support a potential link between the somatotropic system and cerebrospinal fluid regulation. GH treatment has been associated with secondary IIH [44], while therapy with octreotide, a somatostatin analogue, has been reported to improve IIH symptoms [45]. Moreover, the expression of GH and IGF‑1 receptors, as well as somatostatin receptor expression, within the choroid plexus and arachnoid granulations suggest a direct somatotroph modulation of CSF dynamics [17, 46].

Building on the endocrine findings, the structural MRI results in this cohort showed patterns consistent with established imaging markers of raised ICP. Measures of pituitary flattening, sella size, and pituitary gland volume differed only modestly across ICP groups, and the distribution of empty sella classifications showed no significant group differences.

Structural MRI findings in this cohort suggest that intraorbital structures may be more sensitive to ICP-related effects than sellar or pituitary measures [47–50]. The strongest effect was observed for right optic nerve volume in the elevated ICP subgroup (r = 0.903, *p* = 0.005), indicating that the optic nerve compartment may be particularly susceptible to pressure related structural change once ICP exceeds physiological levels. ONS volumes showed similar effect patterns in the normal ICP subgroup, with correlation coefficients narrowly missing significance (left: r = 0.804, *p* = 0.054; right: r = 0.797, *p* = 0.058). This pattern may indicate greater sensitivity of the sheath to variation within the lower pressure range, whereas ON volume appears to reflect more sustained or pronounced ICP elevation. Although the correlations narrowly missed statistical significance, the effect sizes were large, and the *p*-values fell just above the conventional threshold (*p* ≈ 0.05). This near-significant pattern is consistent with the expected biomechanics of raised intracranial pressure: the ONS serves as a distensible CSF-filled compartment and therefore expands early in the course of ICP elevation. The anatomical plausibility, together with the tight correlation coefficients in the normal ICP range, supports the observation that ONS volume is a sensitive but pressure range dependent marker. The lack of significance in the elevated ICP group likely reflects a ceiling effect, whereby the ONS has reached its maximal distension and can no longer track further pressure increases or may reflect sample size limitations or variability in pressure-related structural responses. (see Supplementary Table S4).

Pituitary and sella parameters showed little evidence of pressure dependent variation. The minimal differences across groups and the absence of significant correlations suggest limited responsiveness within the ICP range represented here.

Taken together, the imaging data indicate intraorbital metrics, particularly ON volume, capture ICP-related structural effects more reliably than pituitary or sellar measures. This interpretation aligns with the known biomechanical vulnerability of the optic nerve and may explain the relative insensitivity of midline sellar structures within the pressure range observed.

Taken together, our findings suggest that IIH may involve a pressure related alterations in endocrine regulation accompanied by structural changes detectable on quantitative MRI, particularly within the pituitary region and intraorbital ON compartment. The alignment of some endocrine and imaging patterns suggests an ICP related interaction between hormonal regulation and morphology, although the present data do not allow causal inferences. At the same time, the absence of consistent associations between endocrine parameters and empty sella indicates that morphological compression does not translate into measurable hormonal dysfunction in our dataset, and we therefore refrain from drawing any clinical conclusions from this structural finding.

Several limitations warrant caution. ICP was assessed at a single time point, and the overall sample size was small, resulting in limited statistical power. Most participants were obese, which is a relevant confounder for multiple endocrine axes, and GH status was not examined using stimulation testing. Multivariable adjustments for age, BMI, and pituitary volume in such a small dataset increase the risk of model instability, as illustrated by isolated negative adjusted hormone values. Sensitivity analyses indicated that only large effects were detectable, suggesting that more subtle endocrine-structural relationships may have remained undetected. Variability in several endocrine measures further contributes to uncertainty at the level of individual associations.

Overall, the findings provide preliminary insight into possible neuroendocrine and structural correlates of elevated ICP in IIH and motivate further mechanistic and longitudinal studies with larger cohorts, rather than supporting immediate clinical application.

## Clinical Implications

These exploratory findings suggest potential interactions between ICP, endocrine markers, and structural MRI changes. Given the limited sample size, the variability in endocrine measurements, and the cross-sectional design, the present results cannot inform clinical decision-making or support routine endocrine testing in IIH. Instead, they should be regarded as hypothesis generating and serve primarily to guide the design of future studies with adequate statistical power.
